# Thermosonication of Orange-Carrot Juice Blend: Overall Quality during Refrigerated Storage, and Sensory Acceptance

**DOI:** 10.3390/molecules28052196

**Published:** 2023-02-27

**Authors:** Bárbara Morandi Lepaus, Anna Karoline Pereira de Oliveira Santos, Arthur Favoretti Spaviero, Polliany Strassmann Daud, Jackline Freitas Brilhante de São José

**Affiliations:** 1 Postgraduate Program in Nutrition and Health, Federal University of Espírito Santo, Marechal Campos Avenue, Vitória 29040-090, ES, Brazil; 2 Graduation in Nutrition, Federal University of Espírito Santo, Marechal Campos Avenue, Vitória 29040-090, ES, Brazil; 3 Integrated Health Education Department, Federal University of Espírito Santo, Marechal Campos Avenue, Vitória 29040-090, ES, Brazil

**Keywords:** non-thermal technologies, ultrasound, thermal treatment, bioactive compounds

## Abstract

Ultrasound combined with high temperatures (thermosonication) is an alternative to thermal treatments applied for juice preservation purposes. Blend juices, such as orange-carrot juice, are an interesting option for consumers due to their diversity of unique flavors. The main aim of the present study is to investigate thermosonication’s impact on the overall quality of an orange-carrot juice blend over 22-day storage at 7 °C, in comparison to thermal treatment. Sensory acceptance was assessed on the first storage day. The juice blend was prepared based on using 700 mL of orange juice and 300 g of carrot. The effect of ultrasound treatment at 40, 50, and 60 °C for 5 and 10 min, as well as of thermal treatment at 90 °C for 30 s, on the physicochemical, nutritional, and microbiological quality of the investigated orange-carrot juice blend was tested. Both the ultrasound and the thermal treatment could maintain pH, °Brix, total titratable acidity, total carotenoid content, total phenolic compounds, and the antioxidant capacity of untreated juice samples. All ultrasound treatments improved samples’ brightness and hue value, and made the juice brighter and redder. Only ultrasound treatments at 50 °C/10 min and at 60 °C/10 min have significantly reduced total coliform counts at 35 °C. Thus, they were selected along with untreated juice for sensory analysis, whereas thermal treatment was used for comparison purposes. Thermosonication at 60 °C for 10 min recorded the lowest scores for juice flavor, taste, overall acceptance, and purchase intention. Thermal treatment and ultrasound at 60 °C for 5 min recorded similar scores. Minimal variations in quality parameters were observed over 22-day storage in all treatments. Thermosonication at 60 °C for 5 min has improved samples’ microbiological safety and resulted in good sensorial acceptance. Although thermosonication has the potential to be used in orange-carrot juice processing, further investigations are necessary to enhance its microbial effect on this product.

## 1. Introduction

Juices are a good option to help increase fruit and vegetable intake by the population [1,2]. Based on lifestyle changes observed over the years, fruit and vegetable juice intake has gained popularity and increased worldwide [3]. Orange juice has vitamins, minerals, fiber, and sugars, and it is one of the most consumed juices worldwide. However, it is interesting to encourage the consumption of mixed juices that include fruits and vegetables [4,5]. Carrots hold a high content of bioactive compounds such as carotenoids, as well as provide minerals such as potassium, sodium, and calcium. Thus, blended juices are an interesting option for consumers due to features such as improved sensory and nutritional quality, unique flavors, and variety [4,6,7,8]. Adding carrots to orange juice can contribute to its attractive orange color, as well as help increase its polyphenol and carotenoid contents, and its antioxidant capacity.

Despite the benefits deriving from juice intake, this product spoils quite fast due to microorganisms’ and enzymes’ action, and this process poses a challenge to the juice industry [9,10]. Heat treatments are often applied to juice during processing and storage stages for microbiological control purposes [11,12,13]. Heat application effectiveness in microorganisms’ inactivation relies on changes in the cytoplasm membrane, RNA, and DNA permeability, which, in turn, leads to extravasation of microbial cells’ intracellular content [13,14]. However, undesired changes in juice quality after thermal process application have been reported in the literature. Among them, one finds decreased bioactive compound contents and changes in product color [15,16]. Consequently, interest in new food processing technologies, such as high hydrostatic pressure (HHP), pulsed electric field (PEF), ultraviolet irradiation (UV), and ultrasound (US), has emerged [7,15].

Ultrasound (US), also known as sonication, is an emerging technology capable of generating and emitting high-intensity ultrasonic waves at a frequency higher than 16 kHz. Cavitation—i.e., microbubbles’ formation, increase, and collapse—is US’s primary mechanism. Ultrasonic waves induce the formation of alternate compression and expansion zones. Constant changes in this region lead to bubbles’ increase and collapse because of an inner pressure increase that, at a certain time, becomes higher than the pressure outside them. Free radicals (H^+^ and OH^−^) are formed, diffused to the bubbles’ inner part, and released during collapse [17].

Overall, besides cavitation and free radicals’ formation, physical and chemical phenomena caused by ultrasound comprise agitation, pressure, shock waves, shear forces, microjets, and acoustic transmission [7,18]. Considering the physical and chemical effects of the US, this technology has been tested in food processing carried out for different purposes, such as extraction, homogenization, dispersal, mixing, and decontamination processes, among others [7].

However, the exclusive application (20–25 °C) of this technology in fruit and vegetable juice decontamination processes has shown a low microorganism lethality rate; moreover, it failed to reduce the microbiological charge in juices, even after 30-min exposure to it [19,20]. However, a temperature increase during ultrasonication can lead to increased microbial inactivation and enable achieving the 5-log reduction required by the Food and Drug Administration (FDA) [6,21].

On the other hand, US application in juices should not be limited to ensuring their microbiological quality. This technology must be capable of maintaining juices’ vitamins, anthocyanins, phenolic compounds, and antioxidant capacity, as well as important sensory features, such as flavor and color [22]. Thus, studies focused on investigating the US–heat association (thermosonication) have been conducted to help in improving antimicrobial action in single fruit or vegetable juices. Some authors reported products’ physicochemical quality preservation and good acceptance by consumers [16,23,24].

Despite the aforementioned factors, the impact of this technology on both overall quality during storage and sensory parameters of blended juices still needs to be explored. Thus, the aim of the current study was to assess the effect of thermosonication treatment on orange-carrot juice’s physicochemical (pH, °Brix, total titratable acidity, and color parameters), nutritional quality (total carotenoid content, total phenolic compound, and antioxidant capacity), and microbiological charge (natural contamination) during storage, as well as on its sensory acceptance.

## 2. Results and Discussion

### 2.1. Juice Blend pH, Total Soluble Solids (°Brix), and Total Titratable Acidity (TTA)

All treatments preserved juice blends’ pH, °Brix, and TTA (*p*-value > 0.05). Means recorded for pH, °Brix, and TTA reached 4.11 ± 0.03, 8.46 ± 0.06, and 0.40 ± 0.01, respectively (Table 1). Carrot juice has a naturally high pH, which enables the growth of several spoilage microorganisms during storage time. Its combination with lower pH fruits, such as oranges, is an alternative to help in reducing its pH and providing a less favorable environment for microorganisms’ growth [6,25,26].

**Figure 1:**
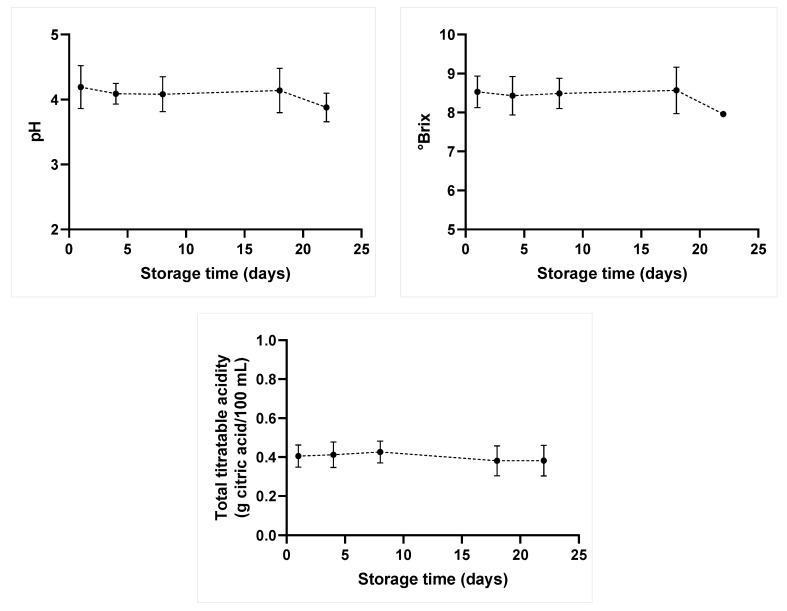
Mean values recorded for pH, °Brix, and total titratable acidity of the orange-carrot juice blend subjected to all treatments, during 22-day storage, at 7 °C. No statistical difference between treatments was observed.

Variations in these parameters were only observed during storage time (*p*-value ≤ 0.05), without significant differences between treatments. This finding indicates that the US has maintained these features throughout storage, similar to those of untreated and thermally treated juices. Linear and quadratic regressions were applied to pH, °Brix, and TTA based on storage time. However, there was no adjustment for a regression model. Then, Figure 1 was elaborated based on using mean values recorded for all treatments, since no statistical difference between treatments was observed during storage time. Juice samples’ pH has shown a small variation between the 1st and the 18th days of storage: there was a higher decrease in this parameter after this period until the end of storage, from 4.14 ± 0.34 to 3.88 ± 0.22. Similar behavior was observed for °Brix values, which reached 7.96 ± 0.03 °Brix on the last storage day. TTA values remained stable over the analyzed storage time.

Hog plum (*Spondias mombin* L.) juice thermosonication (40 kHz, 40–60 °C, 5–30 min) preserved its pH, °Brix, and titratable acidity [24]. Tangerine juice presented a higher °Brix after thermosonication (35 kHz, 60 °C, 5–10 min) than pasteurized (85 °C, for 5 min) and ultrasound (50 °C, for 5 and 10 min) samples [16]. However, US treatment (376 W/10 min/35 °C) increased the pH value from 3.38 to 3.43 in a strawberry-apple-lemon juice blend, although it maintained the °Brix value. This increase was likely associated with the formation of new chemical compounds in the juice due to ultrasound action [8]. Another study [27] has investigated the effect of thermosonication (24 kHz, 60 °C, 15 min) on cashew-apple juice. The authors of the aforementioned study reported a decreased pH (3.60 to 2.98) and an increased residual polyphenol oxidase (PPO) activity (16.87% to 58.4%), after storage at 4 °C, for 90 days.

An acidic environment limits the proliferation of several microorganisms, such as pathogenic or spoilage bacteria; thus, pH is an important factor capable of slowing microbial growth [12]. However, molds and yeasts can survive in media showing this feature [28]. Decreased pH values, in association with a reduced °Brix value after 18-day storage, may indicate higher microbial growth, due to sugars’ fermentation by microorganisms or to high enzymatic changes in juice samples [27].

### 2.2. Color

Color is a critical quality parameter affecting products’ acceptance by consumers. Treatment and storage time have significantly affected orange-carrot juice color (*p*-value ≤ 0.05).

All ultrasound treatments, without differences among them, increased the orange-carrot juice’s brightness (L*) by 3.13% (*p*-value ≤ 0.05), on average, in comparison to untreated samples, after processing. Thermal treatment results were statistically similar to the ones observed for the untreated sample (Table 1).

No difference in hue° values (*p*-value > 0.05) was observed between thermosonication and thermal treatment. Still, the herein adopted treatments significantly decreased the hue° value by 3.16%, on average, in comparison to the control sample (*p*-value ≤ 0.05). All treatments led to increased blended juice chroma values; however, results recorded for the thermal treatment and for US, conducted at 40 and 50 °C (both for 5 min), were statistically similar to the ones recorded for the untreated sample.

US treatment at 35 °C for 10 min has maintained the strawberry-apple-lemon blend juice’s brightness [8], as well as improved this parameter in thermosonicated carrot and hog plum juices [24,29]. Alves et al. [16] have thermosonicated (35 kHz, 750 W; 50, 60 °C; 5 and 10 min) tangerine juice and recorded similar result for the hue° value. The decrease in hue angles gradually changed the sample in a more negative direction and increased the orange color tonality. This occurred due to hydroxyl formation, which favored the red color intensity resulting from phenol aromatic ring hydroxylation [15].

**Figure 2:**
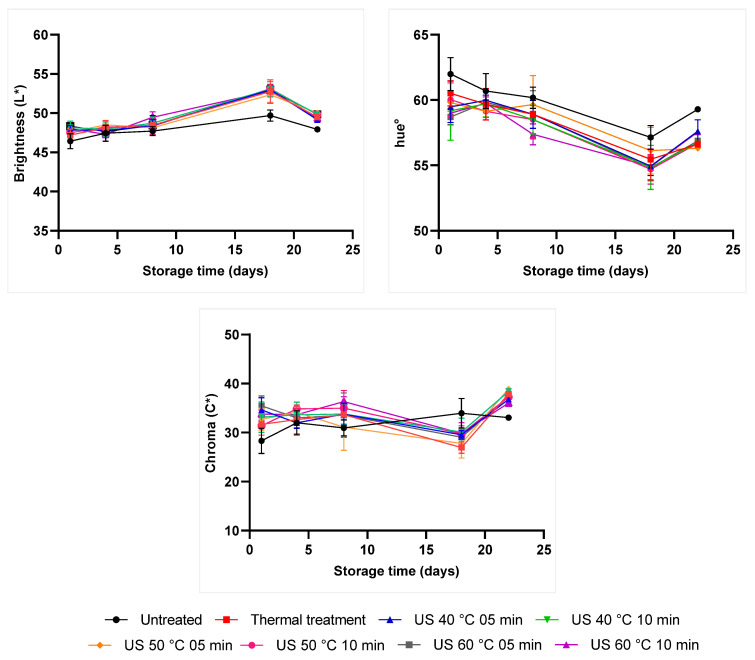
Mean color variation (during 22-day storage) observed for the carrot-orange juice blend subjected to different preservation treatments. US: Ultrasound. Thermal treatment: 90 °C for 30 s.

Color parameters were affected by storage time, with differences between treatments (*p*-value ≤ 0.05). Linear and quadratic regression analyses were applied to the investigated variables based on storage time. However, no significant models and coefficients were obtained; thus, Figure 2 was elaborated to show color behavior during storage time.

All treatments increased juice brightness between the 1st and 18th storage days. This parameter has shown a slight decrease after this period. The untreated sample has shown lower brightness during storage time. The reverse behavior was observed for hue° and chroma values, which increased after 18-day storage (Figure 2). Manothermosonicated apple-carrot juice has shown a significant decrease in L* values over storage at 4 °C for 21 days, in comparison to the untreated sample. On the other hand, no significant differences in redness and yellowness components were observed between juice samples [6].

Preservation methods can lead to cell disruption, favor intracellular compound release, such as pigments, and affect product color [30]. Moreover, enzymatic and non-enzymatic browning after processing can change color parameters. Results in the current study have indicated that ultrasound could improve juice color, since samples turned brighter and redder after US-based processing. On the other hand, the brightness increase during storage time was associated with particle precipitation, a process that made the medium brighter [31]. Electrostatic or Van der Waals forces induced the formation of aggregates comprising small particles, which, in turn, can easily precipitate. The chroma increase between the first and last storage days may indicate yellowness component darkening reactions in the analyzed samples [30], as well as the action of food enzymes, such as peroxidase (POD) and PPO [32].

### 2.3. Total Carotenoids, Total Phenolic Compounds, and Antioxidant Capacity

The bioactive compounds and antioxidant capacity assessment aimed at identifying likely changes in the nutritional quality of juice samples subjected to different preservation treatments, in comparison to the untreated ones (control).

All treatments have maintained the juice blend’s total carotenoids (TC), total phenolic compounds (TPC), and antioxidant capacity, based on the DPPH assay (*p*-value > 0.05) (Table 2). Storage time has significantly affected these parameters (*p*-value ≤ 0.05), although no differences were observed between treatments.

**Figure 3:**
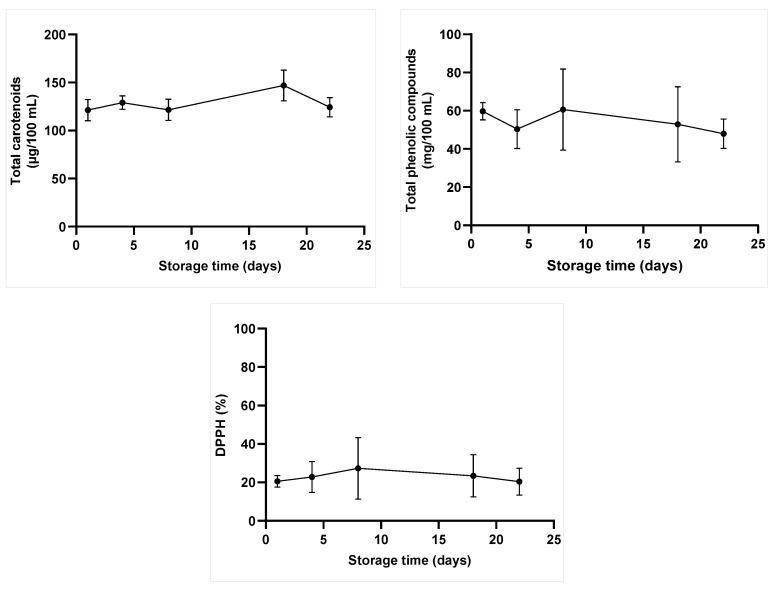
Mean values recorded for total carotenoids, total phenolic compounds, and antioxidant capacity of the orange-carrot juice blend subjected to all treatments, during 22-day storage, at 7 °C. No statistical difference was observed between treatments.

Treatments did not affect (*p*-value > 0.05) TC content on the first day (mean = 121.24 ± 11.23 µg/100 mL). These compounds only underwent more variations on the 18th storage day (Figure 3). Carotenoids are an essential nutrient for human health, as well as the primary pigment in carrot juice [29]. Treatments can disintegrate cellulose in plant walls and favor compound release [33]. These phenomena likely took place in the herein analyzed sample and led to gradual intracellular content release during storage time. Nevertheless, light and oxygen are the main elements accounting for carotenoid degradation [34], a fact that may explain its reduction at certain times. Another hypothesis used to explain carotenoids’ behavior during storage time is that they were subjected to the action of degradative enzymes, such as polyphenol oxidase. Unfortunately, the treatments’ effect on enzyme inactivation was not investigated in the present study. Therefore, future research should focus on performing this assessment.

Treatments have maintained TPC (mean = 59.67 ± 4.51 mg GAE/100 mL) and antioxidant capacity (mean = 20.54% ± 3.95%) after processing (day 1). Modest antioxidant capacity was observed between the 1st and 8th storage days. Minor variations took place over the 22-day storage, without significant differences among treatments (Figure 3). Interestingly, there was no impairment in the blended juice’s nutritional parameters, even after increasing the treatment temperature or processing time.

Similar results were observed after apple-carrot juice manothermosonication (20 kHz; 100% amplitude; 50 and 60 °C; 200 kPa; 30 or 60 s), and no significant differences in TPC and antioxidant capacity were observed between treated samples and the control during storage time [6]. Phenolic compounds are the main elements accounting for antioxidant activity in fruit juices [35]. Some authors observed that an increase in ultrasound treatment time increased TPC; consequently, it also increased antioxidant activity [25,30,35]. It is important to emphasize that bioactive compounds are not formed during food processing. These results can be explained by compounds’ extravasation after cell wall rupture caused by cavitation [36] and by hydroxyl groups formed by phenolic compounds’ aromatic ring [35].

Rodríguez-Rico et al. [37] recently reported a significant increase in total carotenoid concentration after melon juice pasteurization (65 °C, 30 min) or ultrasonication processes (27 and 52 W/cm^2^; 10 and 30 min; 10 ± 2 °C). Moreover, US processing increased DPPH values recorded for melon juice, which reached 45%, in comparison to 39% and 29% recorded for pasteurized and untreated juice samples, respectively. However, samples’ total phenolic content decreased by 33% after US processing. Another study [8] investigated the effect of US (376 W/10 min/35 °C) on a strawberry-apple-lemon juice blend’s TPC and antioxidant capacity. The aforementioned authors observed increases of 7% and 2.4% in TPC and antioxidant capacity, respectively, whereas both parameters remained unchanged after heat treatment at 86 °C, for 1 min. TPC and antioxidant capacity increased in the first two days of storage at 4 °C but decreased after 10 days.

Different results were observed for bioactive compound contents and antioxidant capacity of juices treated with US. These differences took place because the US effect depends on operating conditions (e.g., time, amplitude, frequency, and temperature), as well as on sample features.

Free radicals (e.g., hydrogen and hydroxyls) are often formed during ultrasound treatment. This occurs because samples are exposed to oxidation processes that can increase nutrient degradation, depending on the adopted processing conditions. However, cavitation favors the elimination of dissolved oxygen molecules involved in oxidation processes, and it prevents bioactive compounds from being affected during ultrasonic processing. We did not observe an increase in phenolic compounds’ content and antioxidant capacity in the analyzed samples. The hypothesis raised to explain the herein observed result is that ultrasound may have favored oxidation reactions faster than compounds’ release, which led to no difference between samples. Furthermore, oxidation degradation, and decreased bioactive compounds and antioxidant capacity during storage time, were previously described due to the action of pectin methyl esterase (PME) [38]. Moreover, protein-based polymerization of phenolic compounds can happen in juices and reduce bioactive compound contents during storage time [8].

Although a longer exposure time and plant cell wall rupture may favor several oxidation reactions and compromise parameters, such as sample color [20], all ultrasound treatment conditions applied to the orange-carrot juice blend in the present study maintained the samples’ bioactive compounds and antioxidant capacity.

### 2.4. Microbiological Quality

The herein applied treatments promoted significantly different microbial reductions in the blended juice (*p*-value ≤ 0.05). Table 3 shows the counts of all microorganisms after processing (day 1). None of the samples has shown natural contamination with *E. coli*. The natural microbial count applied to the blended juice showed higher contamination by yeasts and molds (6.54 ± 0.25) than by aerobic mesophilic (4.75 ± 0.05) and coliform bacteria, at 35 °C (3.24 ± 0.66).

Both the thermal and the ultrasound treatments were capable of reducing the aerobic mesophilic bacteria count. However, only US treatments conducted at 50 and 60 °C for 10 min significantly differed from the untreated and thermally treated juice (*p*-value ≤ 0.05), whose aerobic mesophilic bacteria counts decreased to 0.60 and 1.38 log CFU/mL, respectively (Table 3).

With respect to mold and yeast counts, all US treatments, as well as the thermal treatment, were capable of reducing this microbial group (*p*-value ≤ 0.05), in comparison to the untreated sample. However, the highest reduction (1.38 log) in this parameter was observed for the most intense US treatment (60 °C/10 min). Furthermore, only yeast and mold counts conducted after US treatment at 60 °C, for 10 min, reached values statistically different from counts conducted after heat treatment (Table 3).

US treatments conducted at 40 °C, and thermal treatment, slightly reduced coliform counts at 35 °C, and values ranged from 0.24 to 0.58 log CFU/mL. However, they were statistically similar to values recorded for the untreated sample (*p*-value > 0.05). Only US treatment at 50 °C, for 10 min, and at 60 °C, for both investigated times, differed from the thermal treatment (*p*-value ≤ 0.05) in coliforms’ inactivation.

Different from the less intense treatment (40 °C), the increased processing time of ultrasound treatments conducted at 50 and 60 °C enhanced the inactivation of aerobic mesophilic bacteria, yeasts, molds, and coliforms, at 35 °C. The most significant reductions were observed after ultrasound application at 60 °C/10 min in all microbial groups (*p*-value ≤ 0.05). However, none of the assessed treatments achieved the 5-log reduction recommended by the FDA. Ultrasound at 60 °C promoted a reduction of 1.38, 1.38, and 2.15 log CFU/mL for aerobic mesophilic bacteria, molds and yeasts, and coliforms at 35 °C, respectively. To obtain more expressive results, it would be important to increase the treatment time and/or combine ultrasound with another preservation method, but this could increase energy expenditure, extend the processing time, and would affect juice quality.

Some authors have investigated the effect of thermosonication on the microbial quality of fruit and vegetable juices. Alves et al. [16] increased the inactivation of the total plate, molds, and yeasts after thermosonication (35 kHz, 60 °C, for 5 and 10 min) of tangerine juice. Saeeduddin et al. [39] reported total inactivation of total plate count, yeast, and mold in pear juice after ultrasound treatment (20 kHz) at 65 °C for 10 min. Likewise, even after 90 days of storage at 4 °C, Deli et al. [27] reported a total mesophilic aerobic bacteria count and yeast and mold count below 5 log CFU/mL in thermosonicated cashew-apple juice (24 kHz, 60 °C, 15 min).

However, a few studies have demonstrated the effect of thermosonication in blended juices. Feng et al. [8] tested HHP (500 MPa/15 min/20 °C), US (376 W/10 min/35 °C), and heat treatment (HT) (86 °C/1 min) in strawberry-apple-lemon juice blend processing and all treatments demonstrated a similar effect in total aerobic bacteria, molds, yeasts, and coliform count inactivation.

**Figure 4:**
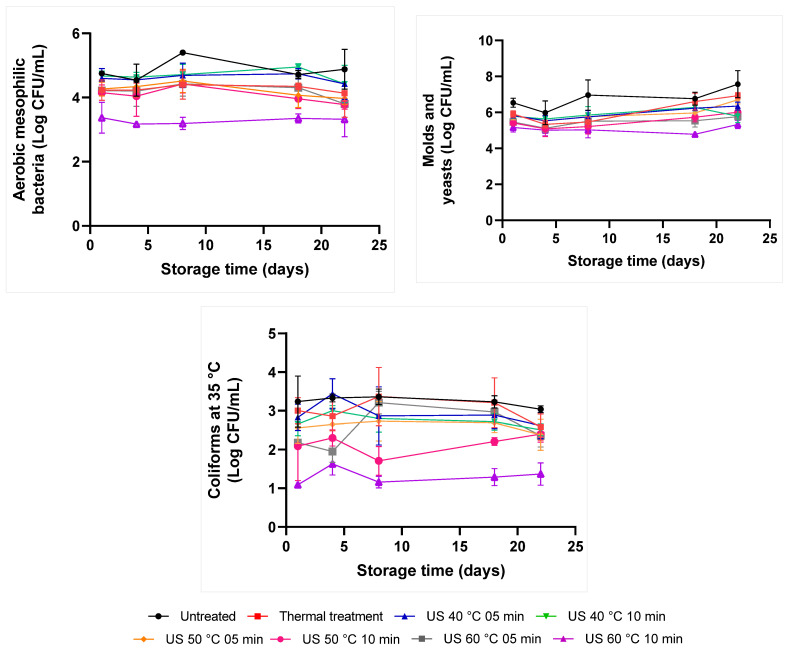
Mean values recorded for microorganisms’ growth during 22-day storage at 7 °C in the orange-carrot juice blend subjected to different preservation treatments. US: Ultrasound. Thermal treatment: 90 °C for 30 s.

Figure 4 demonstrates microbial growth over 22-day storage. The growth of yeasts and molds was more evident over juice storage time, especially after 18 days of storage, which can explain the alterations in pH and °Brix values described in Section 2.1. According to Deli et al. [27], if the microbiological inactivation after preservation treatments is insufficient, sugars present in juices may be fermented by microorganisms, and the total soluble solids content is reduced. The untreated sample showed the highest growth over the storage period, and thermal treatment and ultrasound delayed this proliferation. The pH of the juice may have favored molds and yeasts’ growth since they are more tolerant to acidic mediums than bacteria. Similar results were previously reported [36,40].

Microorganisms’ damage caused by ultrasound is not fully understood; however, it is known that the combination of physical and chemical phenomena contributes to cell membrane breakage, localized heat, free radicals’ release, and cell wall destruction [41]. Molds and yeasts appear to be more resistant to preservation treatments, such as ultrasound cavitation [20,42]. Yeasts have a rigid cell structure, a fact that hinders cell membrane rupture by cavitation [42]. The inactivation of this microbial group takes place mainly due to intracellular protein extravasation, after polysaccharide wall and cell membrane rupture [43]. Besides, exposure to higher temperatures leads to cell membrane weakening and, consequently, to microorganisms’ death [44].

However, microbial inactivation by US depends on aspects such as processing parameters, microorganism type, and food matrix [45]. The application of heat-based treatments can change depending on samples’ features. Mandha et al. [14], for example, observed that the heat transfer time necessary to reach an internal temperature of 80 °C was two-fold higher in mango juice than in watermelon juice. Consequently, the microorganisms’ increase in mango juice was more gradual than that observed in watermelon juice during pasteurization processes. This occurred because watermelon juice presented a higher moisture content capable of absorbing heat faster due to convection.

Therefore, juice viscosity, mainly in mixed juices, may reduce the expected effect of treatments [12,46]; consequently, it may require a longer treatment time or combination with other preservation technologies, for example.

The limited data available in the literature about blended juices treated and stored after thermosonication make it challenging to assess the impact of this technology on the aforementioned product. Assumingly, having two matrices in the juice (orange and carrot) may have compromised heat transfer and cavitation action, requiring more energy. Thus, higher a microorganism inactivation rate was observed in the most intense treatments. Nevertheless, the operating conditions adopted in the present study were not enough to have a sizeable lethal effect on microorganisms, and it may have enabled their growth during storage time. Therefore, future studies should investigate whether an increase in temperature and/or treatment time would affect blended juices’ physicochemical and nutritional parameters. Moreover, the association among ultrasound, temperature, and other technologies, such as microwaves and pressure, among others, should be taken into consideration.

### 2.5. Sensory Acceptance Analysis

Sensory analysis is essential to investigate consumer acceptance in advanced studies focused on applying new food processing technologies. In total, 109 evaluators participated in this research stage, but 6 questionnaires were excluded from the analysis due to filling errors. Thus, 103 participants were taken into consideration for the analysis (*n* = 103). Participants’ ages ranged from 18 to 56 years, with a mean age of 22.8 years. In total, 77.7% (*n* = 80) of participants were female. With respect to intake frequency, 40.9% of participants reported to drink juice on a daily basis, whereas 39.8% of them did so on a weekly basis.

Although all US treatment conditions helped in preserving juice’s physicochemical features, treatments conducted at 60 °C, for 5 and 10 min, were selected for this stage because they were capable of significantly reducing all microbial groups. Untreated and pasteurized juices were also included in this analysis for comparison purposes.

No significant difference (*p*-value > 0.05) in color was observed between samples. The mean color value (7.58 ± 0.07) classification ranged from “I moderately like it” to “I like it very much”, and they indicated that the naked eye could not see the variations observed in instrumental color analysis. Aroma, appearance, consistency, taste, global acceptance, and purchase intention scores were statistically equal between the thermally treated and ultrasound-treated samples (at 60 °C for 5 min). This finding indicates that consumers equally accepted both samples. Small scores recorded for flavor, taste, overall acceptance, and purchase intention were observed for ultrasound-treated juice at 60 °C, for 10 min, and they were represented in the hedonic scale as “I slightly dislike it” and “I did not like or dislike it” (Table 4).

Overall, operation levels applied in food processing conducted with non-thermal technologies (e.g., US, HPP, and cold plasma) were not enough to disrupt covalent bonds. Therefore, compounds accounting for products’ aroma, flavor, and color remained the same [8]. However, the pressure resulting from cavitation in a liquid medium subjected to long-term exposure may lead to undesirable flavors. Aromatic compound degradation is likely associated with extreme physical conditions inside the bubbles during cavitation, as well as with several reactions taking place, either simultaneously or separately [17].

No sensory changes were described in the taste, color, aroma, or consistency of strawberry-apple-lemon juice treated with US, at 35 °C, for 10 min [8]. Sonicated nectars and apple juices (60–120 µm, 3–9 min, 20–60 °C) reached similar evaluation scores for flavor, aroma, odor, and color attributes, in comparison to pasteurized samples, even after 9 min of US treatment [47]. However, a “cooked flavor” was described by panelists after blackberry juice samples were subjected to sonication (25 ± 2 °C) for more than eight minutes [48]. Therefore, there is no consensus about ultrasound’s effect on the sensory features of single-ingredient or mixed juices.

Consumers’ acceptance of products developed based on new technologies can be a challenging task to accomplish. Therefore, it is essential to conduct analyses focused on ensuring products’ sensory aspects based on consumers’ desire, rather than just on assessing their individual microbiological or physicochemical aspects. Each food’s feature can be positively or negatively affected after ultrasound treatment. Thus, it is crucial to assess different operating conditions to help in developing well-accepted products. The highest temperature and the longest exposure time (US 60 °C/10 min) adopted in the current study enhanced microbial inactivation, but they did not preserve the sensory features of the investigated juice.

## 3. Materials and Methods

### 3.1. Experimental Design and Juice Preparation

A completely randomized design was adopted based on following a subdivided plot model conducted in triplicate. Orange (*Citrus sinensis* L. Osbeck) cultivar “Pêra” and carrots (*Daucus carota* L.), without physical damage and free from microbial deterioration visible to the naked eye, were purchased from a local market (Vitória City, Espírito Santo State, Brazil), washed in tap water, and sanitized with 100 mg/L of sodium dichloroisocyanurate (Hidrosteril^®^, Itapevi, São Paulo, Brazil) for 10 min. The orange juice was then extracted (Mondial^®^ Professional E-10 Bivolt—250W, Araçariguama, São Paulo, Brazil). Carrots were peeled and sliced with the aid of a previously sanitized stainless-steel knife. Orange-carrot juice was prepared (700 mL of orange juice and 300 g of carrot) in a blender without the addition of water or any other ingredients to it, according to preliminary tests. Filtered juice samples (400 mL) were stored in previously sterilized glass bottles and subjected to preservation treatments.

### 3.2. Treatments and Storage Study

Samples were subjected to US treatments in bath-ultrasound equipment (Branson^®^, Model CPX3800H, 110 W–40 kHz, Danbury, CT, USA) at 40, 50, and 60 °C, for 5 and 10 min. Ultrasound-treated samples were placed at the center of the equipment (15 and 7.5 cm in length and width, respectively). Thermal treatment was applied in a water bath at 90 °C, for 30 s, for comparison purposes. Samples were removed from the hot water bath and cooled in an ice bath right away. Untreated orange-carrot juice was used as a control.

After the treatments were applied to the investigated samples, juices were stored in glass bottles (previously sterilized at 121 °C, for 15 min), away from light, and kept at 7 ± 1 °C until analysis. Sample analyses were carried out after 1, 4, 8, 18, and 22 days of storage, as described in the following sections.

### 3.3. Physicochemical Aspects and Color Assessment

Sample pH was measured with a calibrated pH meter (Tecnopon^®^, mPA210, Piracicaba, São Paulo, Brazil), based on using 10 mL of juice, at 25 °C. Total °Brix value was determined with the aid of an analogical refractometer (Instrutherm^®^, Freguesia do Ó, São Paulo, Brazil) at 25 °C. Total titratable acidity (TTA) was determined based on using potentiometric titration with 0.1 M sodium hydroxide (NaOH) solution, under agitation, at pH up to 8.2. Sodium (NaOH) volume was used to calculate samples’ TTA, and results were expressed as g of citric acid per 100 mL of juice (g/100 mL) [49].

Color was measured in a previously calibrated spectrophotometric colorimeter used to determine reflectance and transmittance (Konica Minolta^®^, model CR-5, Tokyo, Japan). Brightness (L*), redness (a*), and yellowness (b*) values were recorded, whereas hue angle (*h°*) and chroma (c*) values were calculated based on Equations (1) and (2), respectively [50]:

(1) $$h^{{}^{\circ}}=\tan^{-1}\left(\frac{b^{*}}{a^{*}}\right)$$

(2) $$c^{*}=\sqrt{a^{*}{}^{2}+b^{*}{}^{2}}$$

where a* and b* are values recorded for the juice’s redness and yellowness components, respectively.

### 3.4. Bioactive Compounds and Antioxidant Capacity

Total carotenoid content was extracted and measured based on Rodriguez-Amaya [34], with slight modifications. Accordingly, 3 mL of juice was added to 60 mL of cooled acetone for carotenoid extraction purposes. This mix was homogenized and filtered into a Büchner funnel, with the aid of filter paper. The filtered extract was transferred to a separating flask filled with 50 mL of cooled petroleum ether to enable pigments’ transference from acetone to petroleum ether. Subsequently, 60 mL of distilled water was added to the flask for acetone removal purposes. Extract absorbance was measured based on using quartz cuvettes in a UV/Vis spectrophotometer (Novainstruments^®^ Series 2000–325 A 1000 nm, Piracicaba, São Paulo, Brazil) at 450 nm. Results were calculated based on Equation (3) and expressed as micrograms of β-carotene per 100 mL of blended juice (µg/100 mL).

(3) $$\mathrm{Total}\;\mathrm{carotenoid}\;\left(\mu g/100\;\mathrm{mL}\right)=\frac{A\;\times \;y\;\left(\mathrm{mL}\right)\times 10^{6}}{A_{1\mathrm{cm}}^{1\%}\times 100}\;$$

where A is the sample absorbance at 450 nm, y is the solution volume enabling A absorbance at 450 nm, and $A_{1\mathrm{cm}}^{1\%}$ is the β-carotene absorption coefficient in petroleum ether (2592).

Total phenolic compound extracts and the juice’s antioxidant capacity were analyzed as described by Bloor [51]. Then, 1 mL of homogenized juice was added with 10 mL of a methanol: water solution (60:40 *v*/*v*) and stirred at 180 rpm, at 21 ± 1 °C, for 15 min. After that, the resulting mix was centrifuged at 1413× *g* for 5 min, and the supernatant was transferred to test tubes.

Total phenolic compounds (TPC) were analyzed in spectrophotometer, based on the Folin–Ciocalteau method [52], with slight modifications. An aliquot of 1.5 mL of freshly prepared extract was mixed with 1.5 mL of Folin–Ciocalteau reagent and 1.5 mL of sodium carbonate solution (7.5%). The resulting mixture was homogenized in a vortex mixer (Phoenix Luferco^®^, model AP59, 127/220v, Curitiba, Paraná, Brazil). Sample absorbance was measured at 765 nm (Novainstruments^®^ Series 2000–325 A 1000 nm, Piracicaba, São Paulo, Brazil), after a 2-hour incubation. Results were expressed as milligrams of gallic acid equivalent (GAE) per 100 mL of blended juice (mg GAE/100 mL), based on the calibration curve (y = 26.905x + 0.3352; R^2^ = 0.9979), wherein y is absorbance at 765 nm and x is milligram of GAE per mL of juice.

Antioxidant capacity was tested through the 1.1-diphenyl-2-picrylhydrazyl (DPPH, Sigma Aldrich^®^, São Paulo, São Paulo, Brazil) assay [53]. Aliquots of 400 µL of freshly prepared extract and 4 mL of methanolic DPPH solution were homogenized in a vortex mixer (Phoenix Luferco^®^, model AP59, 127/220v, Curitiba, Paraná, Brazil) and incubated at 21 ± 1 °C, for 1 h. Absorbance decrease was measured at 517 nm, in a UV/Vis spectrophotometer (Novainstruments^®^ Series 2000–325 A 1000 nm, Piracicaba, São Paulo, Brazil). Radical scavenging capacity was expressed as the DPPH radical inhibition rate and calculated based on Equation (4) [37]:

(4) $$\mathrm{DPPH}\;\mathrm{inhibition}\;\left(\%\right)=\left(\frac{A_{\mathrm{control}}-A_{\mathrm{sample}}}{A_{\mathrm{control}}}\right)\times 100$$

where A_control_ and A_sample_ correspond to control and sample absorbance at 517 nm, respectively.

### 3.5. Microbiological Analysis

The juice was homogenized under aseptic conditions, and appropriate dilutions with 0.1% sterile peptone water were prepared for natural juice contamination analysis. Samples were pour-plated in Plate Count Agar (PCA, Himedia^®^, Mumbai, Maharashtra, India) for aerobic mesophilic bacteria enumeration and counted after 48 h at 35 ± 1 °C. Molds and yeasts were analyzed based on the spread-plated technique in Potato Dextrose Agar (PDA, Fluka Analytical^®,^ São Paulo, São Paulo, Brazil), and plates were incubated at 25 ± 1 °C for 5–7 days [54]. Coliforms (at 35 °C) and *Escherichia coli* were analyzed in Petrifilm plates (3M^®^, Maplewood, NJ, USA) and incubated at 35 °C for 48 h. The analysis was carried out in three repetitions, and plating was performed in duplicate. Results were expressed in CFU/mL.

### 3.6. Sensory Analysis

This step was approved by the Research Ethics Committee (Health Sciences Center, Federal University of Espírito Santo; Protocol number 2.569.180). The sensorial analysis was performed based on the sensory acceptance test and the purchase intention. Juices were prepared one day before analysis and kept at 7 ± 1 °C. Tests were conducted in individual booths under white lighting. A nine-point hedonic scale (1: I significantly dislike it, 9: I significantly like it) was applied to assess attributes such as juice samples’ color, aroma, appearance, consistency, taste, and overall acceptance. The nine-point hedonic scale was also used to assess purchase intent (1: I would certainly not buy it, 9: I would certainly buy it). Consumers received approximately 60 mL of each juice sample in transparent plastic cups coded with three random digits, in random order. Participants also received a glass of water to clean their palates before each assessment.

### 3.7. Statistical Analysis

Data were subjected to analysis of variance (ANOVA) to assess the influence of treatment and storage time on the investigated variables. Tukey’s test was used to analyze differences between treatments (*p*-value ≤ 0.05), whereas regression analysis was used to assess significant differences (*p*-value ≤ 0.05) over storage time. Linear and quadratic equation models were tested through regression analysis, based on storage time. Sensory analysis results were subjected to ANOVA and means were compared to each other through the Tukey test (α = 0.05). Data were analyzed based on using SAS^®^ OnDemand for Academics software.

## 4. Conclusions

Although heat treatments are often associated with the loss of sensory aspects in food products, ultrasound and heat treatments performed in the present study could maintain the sensory and nutritional features of the investigated orange-carrot juice. The adopted treatments also had similar effects on these parameters during storage. However, the highest microbial inactivation rate was observed for aerobic mesophilic bacteria, molds, yeasts, and coliforms at 35 °C, after the application of the most intense ultrasound treatment (60 °C, 10 min). On the other hand, this treatment condition resulted in consumers’ lower sensory acceptance of the sample.

Thermosonication conducted at 60 °C, for 5 min, also reduced the sample’s microbial load, maintained the physicochemical features, color, and bioactive compounds, and resulted in good consumer acceptance, compared to untreated and thermal-treated samples. Therefore, the current study has evidenced that thermosonication has the potential to be used as an alternative for orange-carrot juice blend processing since it contributes to products’ physicochemical properties and bioactive compound content maintenance during storage time. Nevertheless, it is necessary to conduct further studies focused on increasing the processing time and/or temperature or combining it with other preservation methods since US could reduce the product’s microbial load. Still, it did not achieve the 5-log reduction recommended by the FDA.

## Figures and Tables

**Table 1:** Means ± standard deviation recorded for variables, such as pH, °Brix, TTA, brightness, and color coordinates, in orange-carrot juice after different preservation treatments, on the first storage day, at 7 °C.

Treatment		pH	°Brix	TTA	Brightness	hue°	Chroma
Untreated		4.17 ± 0.32 ^a^	8.73 ± 0.55 ^a^	0.43 ± 0.06 ^a^	47.84 ± 0.97 ^b^	59.86 ± 1.27 ^a^	31.65 ± 2.57 ^b^
Thermal treatment (90 °C, 30 s)		4.17 ± 0.35 ^a^	8.63 ± 0.57 ^a^	0.38 ± 0.05 ^a^	48.28 ± 0.78 ^b^	58.24 ± 0.96 ^b^	32.55 ± 2.21 ^ab^
US 40 °C	5 min	4.19 ± 0.36 ^a^	8.60 ± 0.39 ^a^	0.43 ± 0.07 ^a^	49.23 ± 1.06 ^a^	58.18 ± 1.19 ^b^	33.33 ± 2.51 ^ab^
	10 min	4.17 ± 0.39 ^a^	8.45 ± 0.41 ^a^	0.38 ± 0.05 ^a^	49.54 ± 0.84 ^a^	57.79 ± 2.25 ^b^	33.81 ± 3.11 ^a^
US 50 °C	5 min	4.18 ± 0.37 ^a^	8.50 ± 0.44 ^a^	0.42 ± 0.08 ^a^	49.30 ± 0.54 ^a^	58.20 ± 0.69 ^b^	32.93 ± 2.23 ^ab^
	10 min	4.25 ± 0.30 ^a^	8.55 ± 0.31 ^a^	0.38 ± 0.05 ^a^	49.40 ± 1.06 ^a^	57.81 ± 1.30 ^b^	33.62 ± 3.25 ^a^
US 60 °C	5 min	4.19 ± 0.29 ^a^	8.37 ± 0.38 ^a^	0.40 ± 0.06 ^a^	49.45 ± 0.43 ^a^	57.83 ± 0.57 ^b^	33.67 ± 2.04 ^a^
	10 min	4.18 ± 0.24 ^a^	8.42 ± 0.43 ^a^	0.43 ± 0.06 ^a^	49.38 ± 0.82 ^a^	57.72 ± 0.89 ^b^	33.91 ± 1.39 ^a^

Means and standard deviation followed by the same superscript letter in the same column did not significantly differ from each other in the Tukey test (*p*-value > 0.05), after three replications. TTA: total titratable acidity (g of citric acid per 100 mL of juice).

**Table 2:** Means ± standard deviation recorded for total carotenoids, total phenolic compounds, and antioxidant capacity in the orange-carrot juice subjected to different preservation treatments, on the first storage day at 7 °C.

Treatment		Total Carotenoids (µg/100 mL)	TPC (GAE/100 mL)	% DPPH
Untreated		110.79 ± 5.21 ^a^	59.62 ± 4.57 ^a^	20.36 ± 4.96 ^a^
Thermal treatment (90 °C, 30 s)		121.33 ± 8.20 ^a^	59.09 ± 4.70 ^a^	19.41 ± 4.42 ^a^
US 40 °C	5 min	117.09 ± 14.85 ^a^	60.19 ± 3.87 ^a^	21.35 ± 3.84 ^a^
	10 min	122.17 ± 14.64 ^a^	59.89 ± 5.01 ^a^	20.87 ± 5.46 ^a^
US 50 °C	5 min	132.20 ± 16.32 ^a^	59.40 ± 4.23 ^a^	20.00 ± 0.35 ^a^
	10 min	121.27 ± 11.08 ^a^	57.99 ± 7.40 ^a^	17.69 ± 6.35 ^a^
US 60 °C	5 min	123.14 ± 7.35 ^a^	60.07 ± 5.96 ^a^	21.37 ± 3.36 ^a^
	10 min	121.91 ± 10.05 ^a^	61.10 ± 5.81 ^a^	23.24 ± 3.37 ^a^

Means and standard deviation followed by the same superscript letter in the same column did not differ by Tukey test (*p*-value > 0.05) after three replications. TPC: total phenolic compounds. GAE: gallic acid equivalent.

**Table 3:** Mean ± standard deviation (log CFU/mL) of natural microbiota counts in the orange-carrot juice subjected to different preservation treatments, on the first storage day, at 7 °C.

Treatment		Log CFU/mL		
		Aerobic Mesophilic Bacteria	Molds and Yeasts	Coliforms at 35 °C
Untreated		4.75 ± 0.05 ^a^	6.54 ± 0.25 ^a^	3.24 ± 0.66 ^a^
Thermal treatment (90 °C, 30 s)		4.23 ± 0.32 ^ab^	5.91 ± 0.18 ^b^	3.00 ± 0.34 ^ab^
Ultrasound 40 °C	5 min	4.60 ± 0.31 ^ab^	5.83 ± 0.11 ^b^	2.83 ± 0.34 ^ab^
	10 min	4.67 ± 0.15 ^ab^	5.79 ± 0.05 ^b^	2.66 ± 0.30 ^abc^
Ultrasound 50 °C	5 min	4.27 ± 0.22 ^ab^	5.81 ± 0.23 ^b^	2.56 ± 0.40 ^bc^
	10 min	4.15 ± 0.24 ^b^	5.41 ± 0.06 ^bc^	2.09 ± 0.89 ^c^
Ultrasound 60 °C	5 min	4.22 ± 0.07 ^ab^	5.46 ± 0.04 ^bc^	2.18 ± 0.75 ^c^
	10 min	3.37 ± 0.48 ^c^	5.16 ± 0.25 ^c^	1.09 ± 0.00 ^d^

Means and standard deviation followed by the same superscript letter in the same column did not significantly differ from each other in the Tukey test (*p*-value > 0.05), after three replications.

**Table 4:** Means and standard deviation recorded for sensory attributes of the orange-carrot juice blend samples on the first storage day (*n* = 103).

Sample	Aroma	Appearance	Consistency	Flavor	Overall Acceptance	Purchase Intent
Untreated	7.29 ± 1.68 ^a^	7.71 ± 1.44 ^a^	7.68 ± 1.38 ^a^	7.56 ± 1.62 ^a^	7.64 ± 1.39 ^a^	7.35 ± 2.10 ^a^
Thermal treatment	6.34 ± 1.80 ^b^	7.57 ± 1.49 ^ab^	7.45 ± 1.52 ^ab^	6.58 ± 2.03 ^b^	6.79 ± 1.82 ^b^	5.94 ± 2.60 ^b^
US 60 °C, 5 min	6.07 ± 2.24 ^b^	7.62 ± 1.74 ^ab^	7.41 ± 1.85 ^ab^	5.85 ± 2.50 ^b^	6.24 ± 2.28 ^b^	5.25 ± 2.74 ^b^
US 60 °C, 10 min	4.21 ± 1.87 ^c^	7.16 ± 1.43 ^b^	6.97 ± 1.61 ^b^	4.14 ± 2.34 ^c^	4.72 ± 1.87 ^c^	3.54 ± 2.82 ^c^

Means and standard deviation followed by the same superscript letter in the same column did not significantly differ from each other in the Tukey test (*p*-value > 0.05). Thermal treatment: 90 °C for 30 s. US: Ultrasound.

## Data Availability

The data supporting this study’s findings are available upon request to the corresponding author.

## Sample Availability

Samples of the compounds are not available from the authors.
